# Genomic evidence of the illumination response mechanism and evolutionary history of magnetotactic bacteria within the Rhodospirillaceae family

**DOI:** 10.1186/s12864-019-5751-9

**Published:** 2019-05-22

**Authors:** Yinzhao Wang, Giorgio Casaburi, Wei Lin, Ying Li, Fengping Wang, Yongxin Pan

**Affiliations:** 1 State Key Laboratory of Microbial Metabolism, School of Life Sciences and Biotechnology, Shanghai Jiao Tong University, Shanghai, China; 2 Institute of Geology and Geophysics, Chinese Academy of Sciences, Beijing, 100029 China; 3 University of Chinese Academy of Sciences, Beijing, 100049 China; 4 Departments of Microbiology and Cell Science, Space Life Sciences Laboratory, University of Florida, Merritt Island, FL 32953 USA; 5 State Key Laboratory of Agrobiotechnology and College of Biological Sciences, China Agricultural University, Beijing, 100193 China

**Keywords:** *Magnetospirillum*, Rhodospirillaceae, Phototrophic, Magnetosome, Oxidative stress

## Abstract

**Background:**

Magnetotactic bacteria (MTB) are ubiquitous in natural aquatic environments. MTB can produce intracellular magnetic particles, navigate along geomagnetic field, and respond to light. However, the potential mechanism by which MTB respond to illumination and their evolutionary relationship with photosynthetic bacteria remain elusive.

**Results:**

We utilized genomes of the well-sequenced genus *Magnetospirillum*, including the newly sequenced MTB strain *Magnetospirillum* sp. XM-1 to perform a comprehensive genomic comparison with phototrophic bacteria within the family Rhodospirillaceae regarding the illumination response mechanism. First, photoreceptor genes were identified in the genomes of both MTB and phototrophic bacteria in the Rhodospirillaceae family, but no photosynthesis genes were found in the MTB genomes. Most of the photoreceptor genes in the MTB genomes from this family encode phytochrome-domain photoreceptors that likely induce red/far-red light phototaxis. Second, illumination also causes damage within the cell, and in Rhodospirillaceae, both MTB and phototrophic bacteria possess complex but similar sets of response and repair genes, such as oxidative stress response, iron homeostasis and DNA repair system genes. Lastly, phylogenomic analysis showed that MTB cluster closely with phototrophic bacteria in this family. One photoheterotrophic genus, *Phaeospirillum*, clustered within and displays high genomic similarity with *Magnetospirillum*. Moreover, the phylogenetic tree topologies of magnetosome synthesis genes in MTB and photosynthesis genes in phototrophic bacteria from the Rhodospirillaceae family were reasonably congruent with the phylogenomic tree, suggesting that these two traits were most likely vertically transferred during the evolution of their lineages.

**Conclusion:**

Our new genomic data indicate that MTB and phototrophic bacteria within the family Rhodospirillaceae possess diversified photoreceptors that may be responsible for phototaxis. Their genomes also contain comprehensive stress response genes to mediate the negative effects caused by illumination. Based on phylogenetic studies, most of MTB and phototrophic bacteria in the Rhodospirillaceae family evolved vertically with magnetosome synthesis and photosynthesis genes. The ancestor of Rhodospirillaceae was likely a magnetotactic phototrophic bacteria, however, gain or loss of magnetotaxis and phototrophic abilities might have occurred during the evolution of ancestral Rhodospirillaceae lineages.

**Electronic supplementary material:**

The online version of this article (10.1186/s12864-019-5751-9) contains supplementary material, which is available to authorized users.

## Background

Magnetotactic bacteria (MTB) are a collection of microbes that produce intercellular, nanosized and chain-arranged magnetite (Fe_3_O_4_) or greigite (Fe_3_S_4_) crystals called magnetosomes [1–3]. Magnetosome biomineralization is a highly organized process under the strict genetic control of a cluster of genes named the magnetosome gene cluster (MGC) [3, 4]. Magnetosomes enable MTB to navigate along the Earth’s magnetic field, usually down to the sediment near the oxic-anoxic transition zone (OATZ), and this ability is known as magnetotaxis [4–8]. However, there is convincing evidence that some MTB are able to actively respond to different wavelengths of light, including the ultraviolet spectrum [9–17]. For example, several MTB, such as multicellular magnetotactic prokaryotes (MMPs) and the marine coccus strain MC-1, showed a negative response to light [9–14]. It has been suggested that the photosensing domain protein genes in uncultured MMP genomes are involved in phototactic movements and that MMPs may avoid damage or lethality caused by long-term irradiation from light and ultraviolet radiation [10, 14]. Moreover, the light wavelength-dependent MMP motility and magnetic sensibility changes have also been discovered [12].

Illumination also influences the growth and magnetosome synthesis of cultured MTB [15–17]. Recent research found that *Magnetospirillum magneticum* AMB-1, a well-studied MTB strain, was able to not only swim towards visible light [15] but also increase magnetosome synthesis and upregulate stress-related genes [16]. Ultraviolet illumination can delay AMB-1 cell growth and induce both cellular and DNA damage [17]. These phenomena demonstrate an intriguing topic regarding the artificial control of MTB motility and growth by magnetism and photons in bioengineering. However, the mechanism by which MTB respond to illumination and potentially cope with the damage induced by illumination (both visible and ultraviolet) remain unknown.

MTB are distributed in five bacterial phyla, namely, Proteobacteria, Nitrospirae, Omnitrophica, Latescibacteria and Planctomycetes [18, 19]. *Magnetospirillum* spp. are a group of facultative anaerobic microaerophiles that are members of a well-studied and sequenced genus belonging to the family Rhodospirillaceae in the class Alphaproteobacteria [1, 2, 20].

Rhodospirillaceae, so-called purple nonsulfur bacteria, encompass a total of 34 genera within the order Rhodospirillales and has the type genus *Rhodospirillum*, which is capable of photosynthesis [20]. Many members of this family can synthesize bacteriochlorophyll a and carotenoids and grow photoheterotrophically under anoxic conditions in light while chemoheterotrophically in darkness. Despite the disparate life styles of magnetotactic *Magnetospirillum* spp. and phototrophic *Rhodospirillum* spp., they were closely clustered based on 16S rRNA gene phylogenetic trees [20, 21].

An important discovery was made by Kolinko et al. via the transfer of magnetosome biomineralization genes from *Magnetospirillum gryphiswaldense* to the photosynthetic model organism *Rhodospirillum rubrum*, suggesting that both bacteria have the ability to host MGCs in their genomes and to provide a similar intracellular microenvironment for synthesizing magnetosomes [22]. Therefore, the close relationship between these MTB and phototrophic bacteria motivated us to question whether the common ancestor of MTB and phototrophic bacteria within the family Rhodospirillaceae possessed both abilities. Two hypotheses regarding MTB evolution remain hotly debated. One hypothesis is that the common ancestor was able to produce magnetosomes, but some bacteria lost this trait due to environmental differentiation or physiological pressures [21–24]. The other hypothesis is that the magnetosome synthesis genes were inherited via horizontal gene transfer (HGT) [25–27].

To detect the possible relationship between magnetotactic bacteria and other members within the family Rhodospirillaceae, we performed a comprehensive genomic comparison between magnetosome synthesis and photosynthetic bacteria within this family. First, we attempted to reveal potential photoresponse mechanisms via the identification of photosensitive genes in their genomes; second, we compared their gene similarities and differences in stress-related systems that may be induced by illumination; and finally, we discussed the possible evolutionary history of MTB and phototrophic bacteria in Rhodospirillaceae.

## Results

### Genomic features and magnetosome gene clusters of *Magnetospirillum* sp. XM-1

*Magnetospirillum* sp. XM-1 was isolated from Xi’an city moat, China. This strain can synthesize chain-arranged magnetite magnetosomes within the cell [28]. The XM-1 genome comprises one circular chromosome of 4,825,187 bp and one plasmid of 167,290 bp with a GC content of 65.64 and 66.48%, respectively (Additional file 1: Figure S1), and is most closely related to the completely sequenced genome of *Magnetospirillum magneticum* AMB-1 strain [29]. The 16S rRNA gene of XM-1 shows a high identity (> 99%) with the strain AMB-1, but the whole-genome colinearity analysis reveals that the XM-1 genome displays multiple rearrangements and insertions when compared with the AMB-1 genome (Additional file 1: Figure S2). The average nucleotide identity (ANI) and average amino acid identity (AAI) of the two complete genomes are 86.7 and 73.2%, respectively, which are lower than the defined species cut-off values (> 95–96% for ANI and > 95% for AAI), indicating that XM-1 represents a novel MTB species within the genus *Magnetospirillum*.

**Fig. 1:**
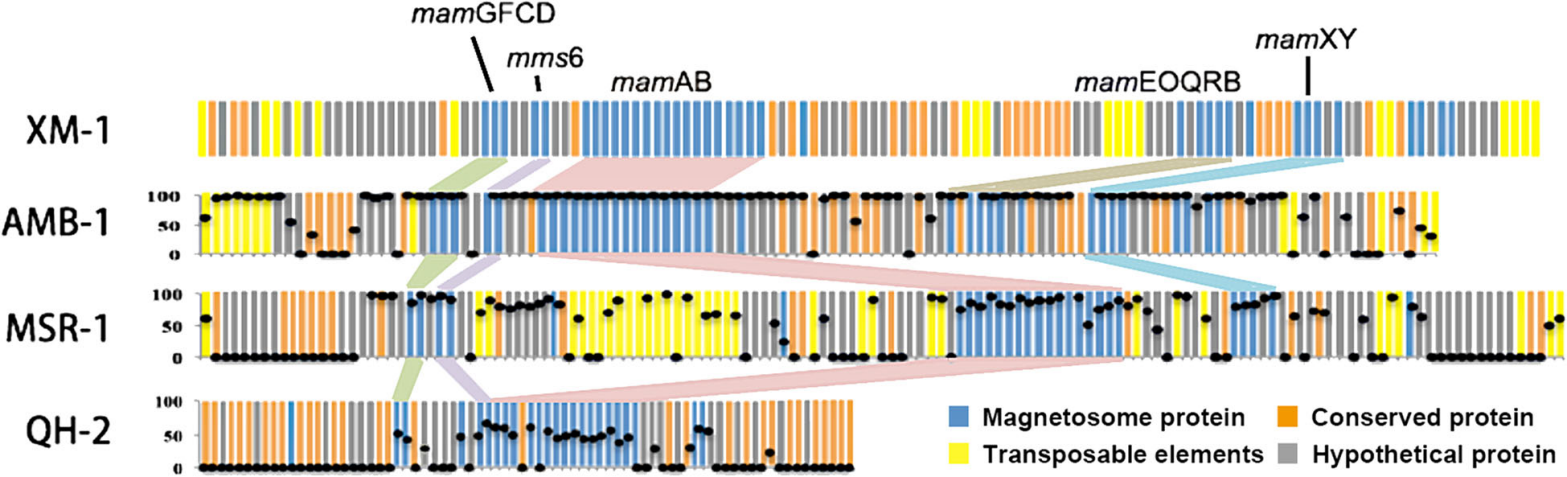
Magnetosome gene cluster comparison between *Magnetospirillum* sp. XM-1 and *Magnetospirillum* strains AMB-1, MSR-1 and *Magnetospira* sp. QH-2, respectively. The black dots represent the highest identities between protein sequences from the XM-1 MGC and MGCs from the other three MTB strains using the BLASTp algorithm. The conserved magnetosome synthesis genes are marked above

The genomic information of the other three published and completely sequenced MTB strains, i.e., *Magnetospirillum magneticum* AMB-1 [30], *Magnetospirillum gryphiswaldense* MSR-1 [31], and *Magnetospira* sp. QH-2 [32], are listed in Additional file 2: Table S1. The genome sizes of the four strains are approximately 4.02 Mb to 4.97 Mb; however, only XM-1 and QH-2 contain plasmids. All four stains contain MGCs in their chromosomes. The magnetosome synthesis gene operons *mam*GFCD, *mms*6, *mam*AB, *mam*EOQRB and *mam*XY are present in their genomic regions, which are also composed of multiple conserved and hypothetical proteins and transposable element genes. All canonical magnetosome genes in the MGC show high identities between XM-1 and the other three MGCs from AMB-1, MSR-1 and QH-2 (Fig. 1). Except for the known magnetosome genes, many genes encoding hypothetical proteins are also conserved in all four MGCs, possibly having critical functions in magnetosome synthesis or iron regulation. Several transposable element genes display high identities among XM-1, AMB-1 and MSR-1, indicating that the entire MGC region may have been inherited vertically from a common ancestor or horizontally transferred from an MTB donor to their ancestral lineage before diversification.

### The *Magnetospirillum* sp. XM-1 genome displays high similarity with the phototrophic bacteria *Phaeospirillum* spp. genomes

The genome of *Magnetospirillum* sp. XM-1 also shares high identity to the genomes of nonmagnetotactic phototrophic bacteria *Phaeospirillum molischianum* DSM 120 and *Phaeospirillum fulvum* MGU-K5, with AAIs of 70.82 and 70.8%, respectively. These AAI values are higher than that between XM-1 and MSR-1 (AAI: 67.88%), both of which are from the same genus, *Magnetospirillum*. These results are consistent with previous phylogenetic analyses based on the 16S rRNA gene, which showed that *Phaeospirillum* spp. branched within the MTB genus *Magnetospirillum* [21, 29, 31].

**Fig. 2:**
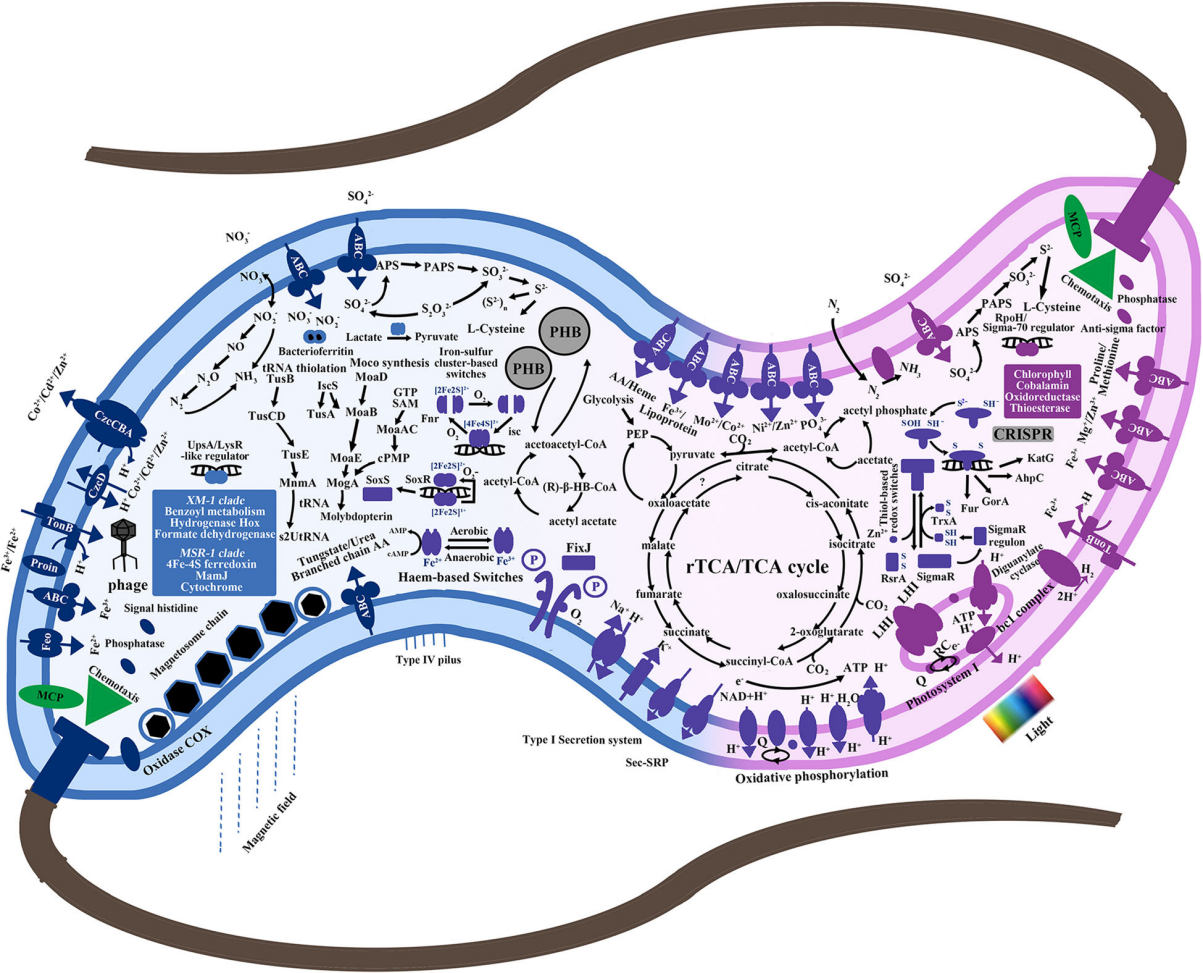
Comparison of metabolic reconstruction maps between *Magnetospirillum* sp. XM-1 (components colored in blue) and *Phaeospirillum* spp. (components colored in pink). The components that are shared by both genera are colored in purple. Please see Additional file 4: Table S3 for details

To better understand the possible relationship between MTB and phototrophic bacteria, the metabolic potentials (Fig. 2) of the newly sequenced *Magnetospirillum* sp. XM-1 genome were compared with the closely related photosynthetic bacteria in the genus *Phaeospirillum* (*Phaeospirillum molischianum* DSM 120 and *Phaeospirillum fulvum* MGU-K5) [33, 34]. The carbon metabolism of *Magnetospirillum* sp. XM-1 and *Phaeospirillum* spp. is similar, and both of their genomes possess genes involved in carbohydrate utilization, and this has been demonstrated by the heterotrophic growth of these strains with sole carbon sources such as acetate, fumarate and succinate in microaerobic conditions [28, 35–37]. For autotrophic growth, nearly all *Magnetospirillum* genomes possess genes of the reductive tricarboxylic acid (rTCA) cycle and the reductive pentose phosphate pathway, i.e., the Calvin–Benson–Bassham (CBB) cycle to fix CO_2_; however, only strains from the genus *Magnetospirillum* were experimentally verified to be capable of carbon fixation with the addition of NaHCO_3_ as a carbon source and Na_2_S_2_O_3_ as an electron donor [28, 38]. In contrast, several autotrophic growth tests on the genus *Phaeospirillum* showed that both photolithoautotrophy (anaerobic with light, electron donor: Na_2_S, Na_2_S_2_O_3_ and carbon source: NaHCO_3_) and chemolithoautotrophy (aerobic in darkness with electron donor: Na_2_S_2_O_3_ and carbon source: NaHCO_3_) could not be conducted [35, 36]. For nitrogen metabolism, nitrogenase genes have been found in both genera, and atmospheric dinitrogen can act as the sole nitrogen source during growth in multiple experimental tests [28, 29, 33, 34, 37–39]. However, only *Magnetospirillum* can use other forms of inorganic nitrogen sources such as nitrate, nitrite and ammonium through the denitrification pathway [28, 40], while *Phaeospirillum* can use ammonium, glutamate and urea as nitrogen sources [35, 36]. For sulfur metabolism, the assimilatory sulfate reduction pathway and dissimilatory sulfite reductase genes (*dsr*) are present in both *Magnetospirillum* and *Phaeospirillum*, which have been tested by the addition of sulfate, sulfite and cysteine as sulfur sources [28, 37, 40].

### Genomic evidence of the photoresponse mechanism in MTB and phototrophic bacteria in the family Rhodospirillaceae

To make a comprehensive comparison, an additional 7 near-complete MTB genomes from Rhodospirillaceae were chosen for this study, namely, *Magnetovibrio blakemorei* MV-1 [41], *Magnetospirillum caucaseum* SO-1 [42], *Magnetospirillum magnetotacticum* MS-1 [43], *Magnetospirillum marisnigri* SP-1 [44], *Magnetospirillum moscoviense* BB-1 [44], *Magnetospirillum* sp. 64–120 [45] and *Terasakiella* sp. PR1 [46]. We did not include the analysis of *Magnetococcus marinus* MC-1 [47], *Magnetococcus massalia* MO-1 [48] or *Magnetofaba australis* IT-1 [49] because a recent study has shown that MO-1, together with MC-1 and IT-1, may comprise a new class of Etaproteobacteria and represent the earliest branching lineage in Alphaproteobacteria [48, 50]. Other selected genomes within the family Rhodospirillaceae, including nearly all sequenced phototrophic and nonphototrophic nonmagnetic bacteria, are approximately 3 Mb to 7 Mb in size (Additional file 3: Table S2, downloaded from NCBI prokaryote genomic database before Jan, 2017), with the exception of *Endolissoclinum faulkneri* L2 and L5 (~ 2 Mb), which are thought to have a parasitic lifestyle. As expected, no magnetosome genes were found in the genomes of phototrophic bacteria, whereas no photosynthesis genes were identified in the MTB genomes. Nevertheless, all bacteria within Rhodospirillaceae contain distinct photoreceptor genes and stress response genes such as oxidative stress genes, iron homeostatic genes and DNA damage repair genes.

#### Photoreceptors in MTB from the family Rhodospirillaceae

**Fig. 3:**
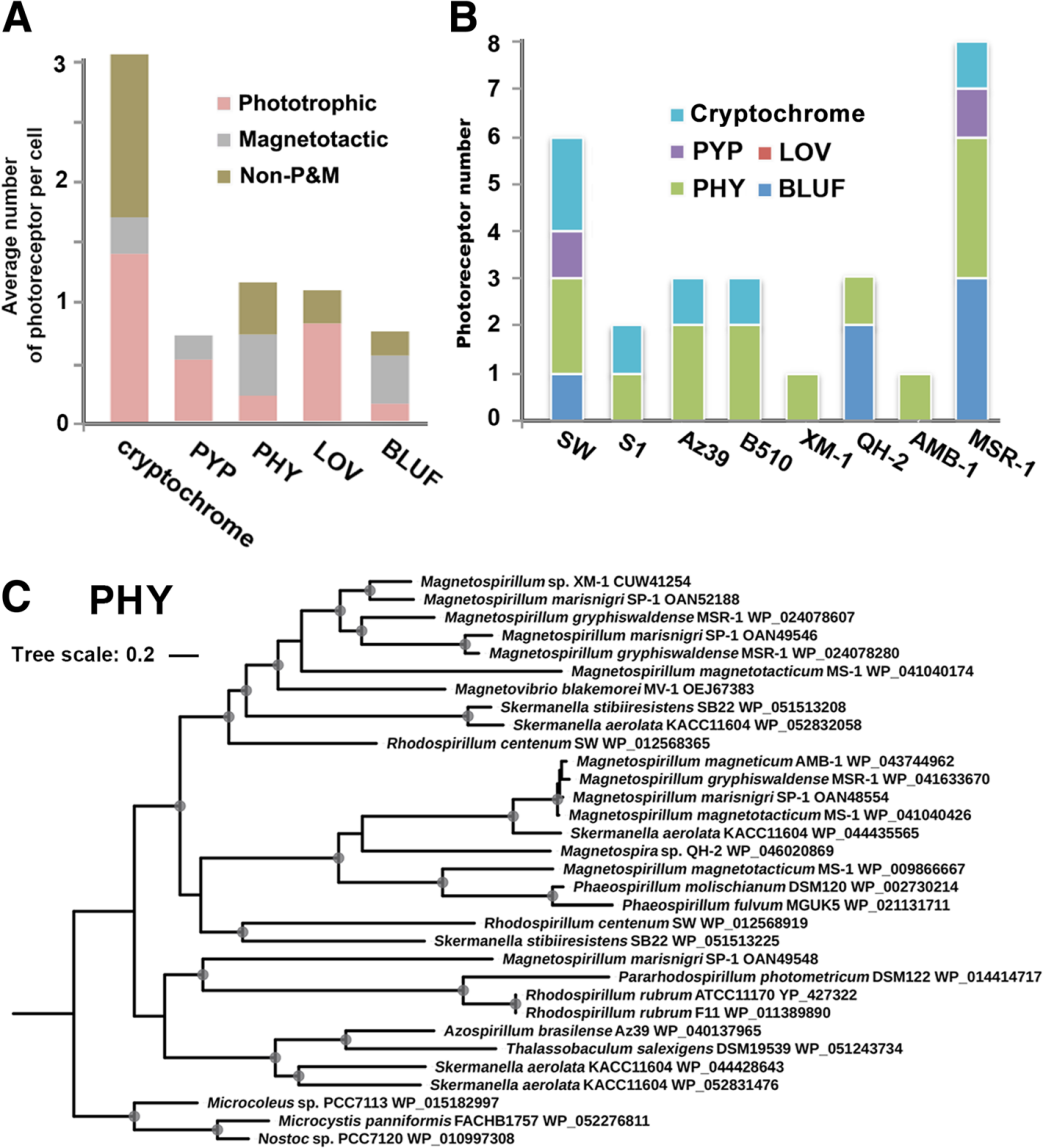
(**a**) Average number of photoreceptors per cell in MTB, phototrophic bacteria and nonphototrophic nonmagnetotactic bacteria within the family Rhodospirillaceae. Photoreceptors were sorted by the following types: cryptochrome, PYP, PHP, LOV and BLUF domain-containing proteins. (Note: the number represents the total number of a certain type of photoreceptor divided by the bacterial genome number). (**b**) Photoreceptors predicted from the completely sequenced MTB strains XM-1, MSR-1, AMB-1, QH-2, phototrophic bacteria strains S1 and SW and nonphototrophic nonmagnetotactic bacteria Az39 and B510. (**c**) Phylogenetic trees based on the PHY domain-containing photosensor protein sequences from the genomes of the Rhodospirillaceae family

Diverse photosensitive domain-containing proteins, including blue light-sensitive sensors of blue light using FAD (BLUF), light-oxygen-voltage photoreceptor (LOV) domain-containing proteins, red/far red light-sensitive phytochromes (PHYs), photoactive yellow proteins (PYPs) and cryptochrome domain-containing protein genes, were found in MTB and other genomes of members in the Rhodospirillaceae family (Fig. 3). No obvious pattern in the category and number of photoreceptors was found among MTB, phototrophic and nonphototrophic nonmagnetotactic bacteria (Fig. 3a), i.e., different bacterial strains contained different numbers and types of photoreceptors regardless of classification as MTB or phototrophic bacteria (Fig. 3b, here we displayed eight representative strains).

Specifically, nearly all MTB and *Phaeospirillum* strains as well as many other phototrophic and nonphototrophic nonmagnetic bacteria within the family Rhodospirillaceae possess one or more PHY domain photoreceptor genes, which is a known sensor for red/far-red light. The MTB strains MSR-1, SP-1 and BB-1 from *Magnetospirillum* and phototrophic bacteria from the genera *Phaeospirillum*, *Skermanella* and *Rhodocista* possess PYP domain photoreceptor genes. These photosensitive domains usually are coupled with homology of the amino-acid motif GGDEF (Gly-Gly-Asp-Glu-Phe) and conversed residues of the EAL domain proteins, which might be involved in light-dependent gene regulation, although their exact functions in MTB have not yet been verified by laboratory experiments. Some strains in Rhodospirillaceae, such as *Magnetospirillum* MSR-1 and BB-1 and *Magnetospira* sp. QH-2, and the genera *Azospirillum* and *Caenispirillum* contain BLUF domain protein genes, while no LOV domain protein-coding genes have been identified in MTB. Intriguingly, cryptochrome domain-containing protein-coding genes, which encode deoxyribodipyrimidine photolyase, are present in nearly all of the Rhodospirillaceae non-MTB strains and MTB MSR-1, SP-1 and BB-1 but are absent in other MTB strains.

In general, the results above show that MTB in Rhodospirillaceae contain distinct types of photoreceptor genes that are also shared by phototrophic bacteria and nonphototrophic nonmagnetic bacteria. No preference for a certain photoreceptor was found among the Rhodospirillaceae bacteria. For example, the phylogenetic analysis of the PHY domain photoreceptor, which is most widely spread in MTB and other members in Rhodospirillaceae, indicates that the photoreceptor is not conserved within MTB or phototrophic bacteria. MTB from the same genus even carry different subtypes of the PHY photoreceptor gene, as revealed in the topology of the phylogenetic tree (Fig. 3c). We assume that photoreceptor genes in Rhodospirillaceae were loosely selected or underwent active horizontal gene transfer during their early evolutionary processes. Although most of these bacteria have been experimentally shown to actively or negatively react with illumination, their physiological functions that are induced by the photoresponse still need to be systematically studied.

#### Stress response pathway comparison within the family Rhodospirillaceae

Since illumination can cause cell damage and even cell death, we specifically focused on oxidative stress, iron homeostasis and DNA damage repair between the MTB and non-MTB within Rhodospirillaceae. The representative completely sequenced genomes of the MTB strains XM-1, AMB-1, MSR-1 and QH-2, phototrophic strains S1 and SW, and nonmagnetic and nonphototrophic strains Az39 and B510 were used to display the general stress response system (the oxidative-related genes of all Rhodospirillaceae members are listed in Additional file 5: Table S4).

**Fig. 4:**
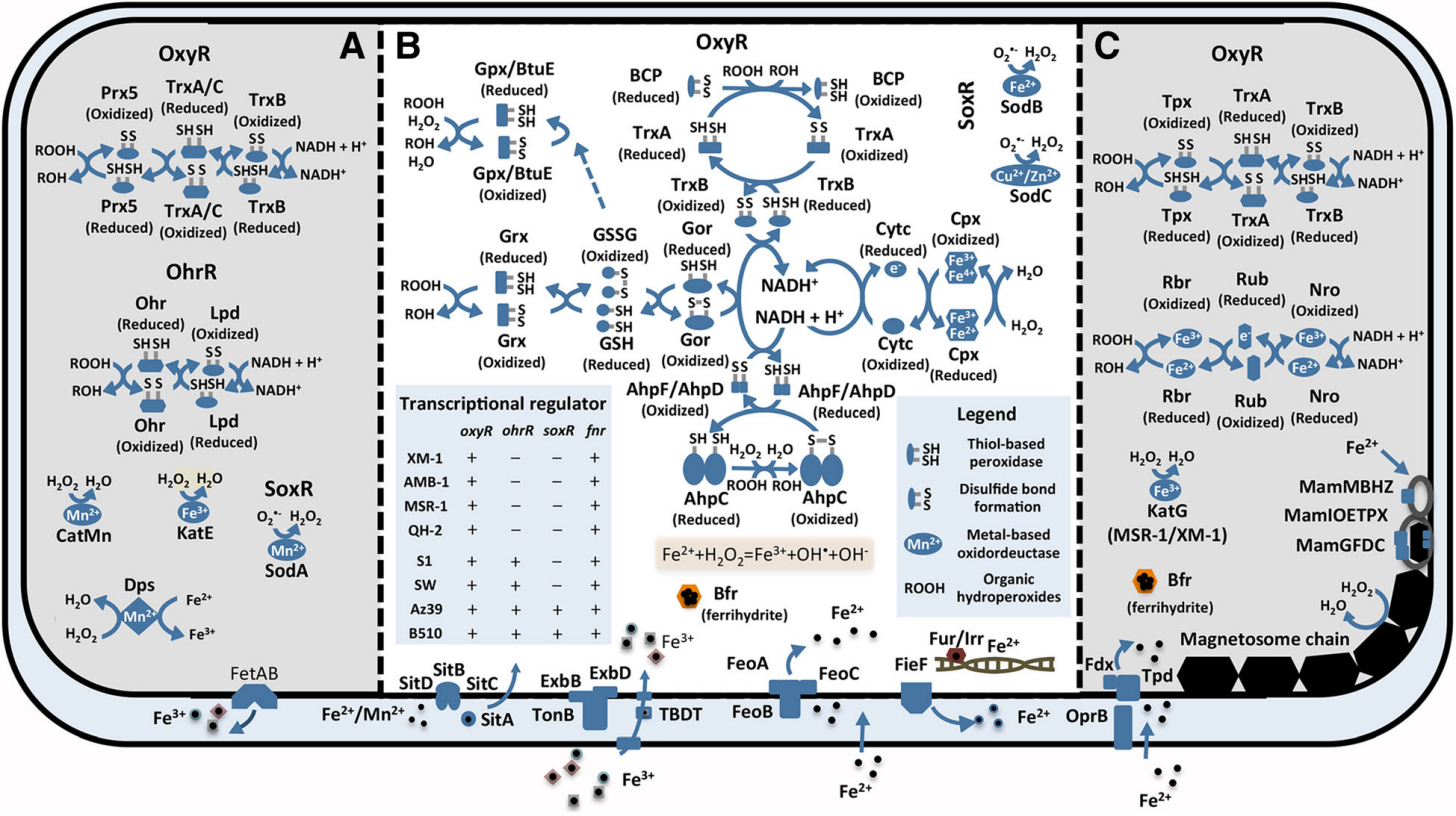
Comparison of oxidative stress systems between MTB and non-MTB within the family Rhodospirillaceae*.* (**a**), (**b**) and (**c**) are the oxidative stress response and elimination processes that are mostly present in the genomes of only non-MTB, both MTB and non-MTB and only MTB, respectively. Iron transport systems from Rhodospirillaceae are displayed in the cell periplasmic space at the bottom of the diagram. Please see Additional file 5: Table S4 for details

In the Rhodospirillaceae family, MTB and non-MTB contain complex systems to cope with intracellular oxidative stress (Fig. 4). Genes encoding the oxidative stress regulatory protein OxyR and redox-sensitive transcriptional activator SoxR-regulated thiol-based stress response systems, namely, the thioredoxin peroxidase BCP, alkyl hydroperoxide reductase peroxiredoxin AhpC, glutaredoxin Grx and glutathione peroxidase Gpx, together with the metal-based cytochrome C peroxidase Cpx and superoxide dismutase SodB/C, were found in nearly all studied genomes and considered to be the main oxidative balancing mechanisms. Nevertheless, MTB and non-MTB also have some different preferences for genes related to stress response systems; for example, the MTB genomes contain the 2-Cys peroxiredoxin Tpx and rubrerythrin Rbr for peroxide stress, while the non-MTB genomes possess the peroxiredoxin Prx5 system. Compared with non-MTB, nearly all MTB lack the genes encoding the organic hydroperoxide responding Cys-based redox sensor OhrR and SoxR proteins. Most non-MTB genomes contain the catalase CatMn, KatE and DNA protection protein Dps genes to scavenge hydrogen peroxide, while MTBs contain fewer enzymes (catalase KatG) for H_2_O_2_ elimination. However, several studies have shown that the magnetosome itself was able to catalyze H_2_O_2_ to H_2_O and O_2_ [51, 52].

Oxidative stress is also closely associated with iron homeostasis because ferrous iron can generate reactive oxygen species through the Fenton reaction (Additional file 1: Figure S3). Most MTB and non-MTB possess genes encoding multiple iron transport systems, including the Feo-dependent ferrous transport system, TonB-dependent ferric transport system, ferrous efflux FieF, iron storage Bfr and iron regulation factor Fur/Irr (Additional file 1: Figure S3). Compared to the non-MTB genomes, the MTB genomes usually contain two or more Feo-dependent ferrous transport system genes, and one *feo* gene operon has been identified within MGC. Nearly all members of the Rhodospirillaceae family contain genes encoding the TonB-dependent ferric transport system, but these genes are absent in *Magnetospira* sp. QH-2. The QH-2 genome contains more genes encoding iron export proteins, such as FetAB and FieF, than iron import proteins. The Tpd-like high-affinity ferrous iron transporter genes are present in the MTB genomes (except for QH-2) along with several copies (usually two) of the Btr iron storage protein genes. These iron-specific genes are vital for iron acquisition and magnetosome synthesis.

Illumination can also induce DNA damage through oxidative stresses or directly react with DNA molecules. Nearly all Rhodospirillaceae strains contain genes encoding DNA damage repair regulators such as LexA and RecA, the recombination system RecBFGJNOQR and RuvABC (Additional file 1: Figure S4). Compared to non-MTB, MTB within the Rhodospirillaceae family generally lack genes encoding enzymes involved in direct reversal (AlkB, Dcd) and base excision repair (AlkA, Mug, Nfi). However, most MTB strains contain genes for methylation systems, such as Dam and Dcm, and double copy genes of single-stranded binding protein Ssb, which is essential for DNA replication and repair. Interestingly, the newly isolated strain XM-1 has three copies of the translation error-prone DNA polymerase V UmuCD genes, MSR-1 and AMB-1 possess two and one copy of UmuCD, respectively, while few homologs were found in non-MTB strains. Ultraviolet radiation can also cause pyrimidine dimers, which can be repaired by the nucleotide excision repair (NER) excinuclease system. Nearly all studied strains contain genes of NER excinuclease systems UvrABCD, while only non-MTB and MTB MSR-1, BB-1 and SP-1 possess the deoxyribodipyrimidine photolyase Phr gene.

In general, stress response pathways are universally shared by all members of the Rhodospirillaceae family. They contain complex but similar systems to cope with intracellular oxidative stress and balance iron homeostatic conditions. Although these bacteria also contain comprehensive DNA damage repair genes in their genomes, MTB in the Rhodospirillaceae family overall contain fewer DNA damage repair genes when compared with non-MTB as revealed by the current database (Additional file 1: Figure S5).

### Phylogenomic analyses of the relationship between MTB and phototrophic bacteria in the family Rhodospirillaceae

**Fig. 5:**
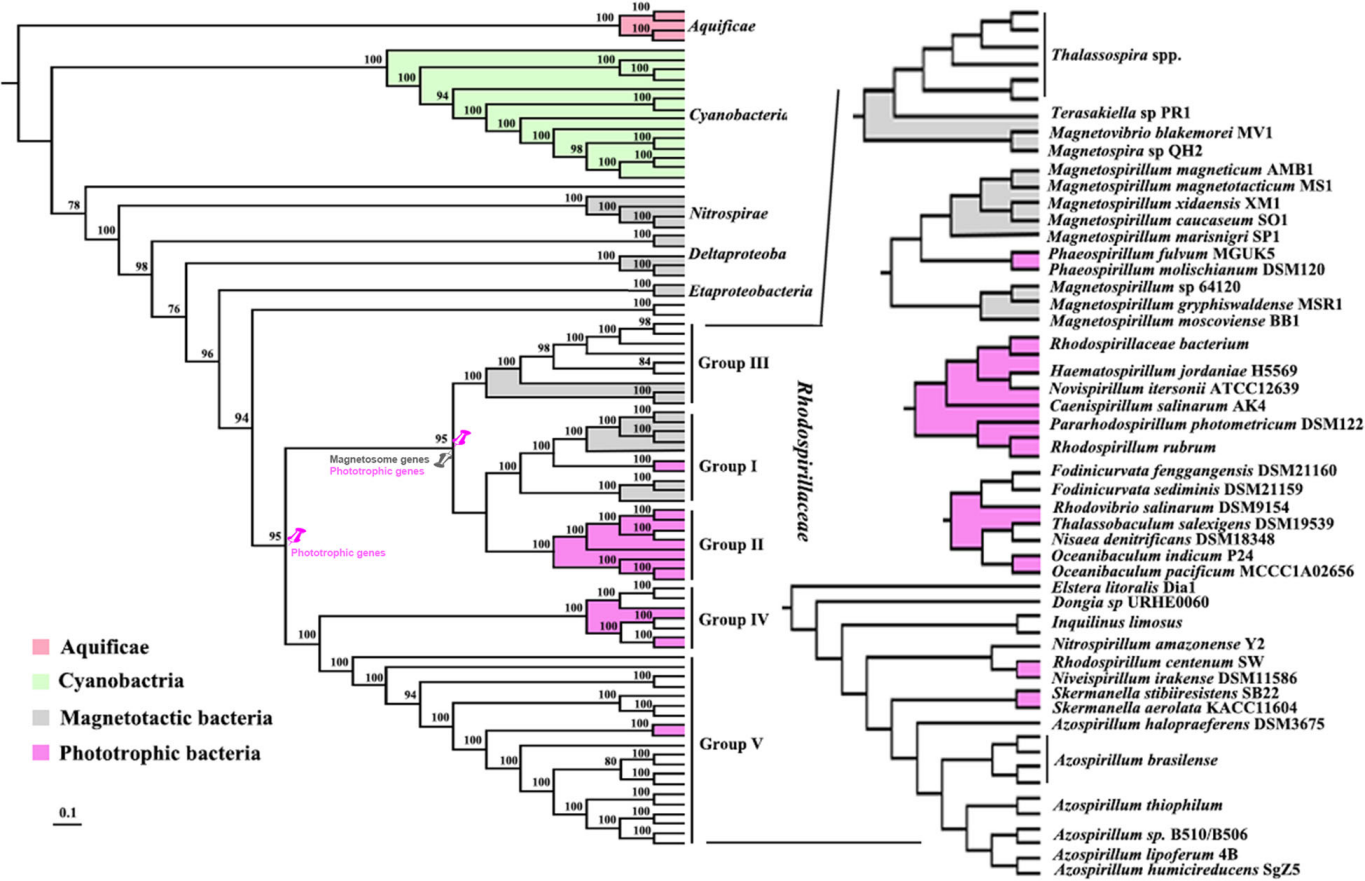
Phylogenomic tree of genomes from the family Rhodospirillaceae using the PhyloPhlAn method. Bacterial genomes from Aquificae and Cyanobacteria were also selected as out groups. Different colors represent distinct features within the Rhodospirillaceae family*.* The gray and pink colors represent magnetotactic and phototrophic bacteria, respectively. Five groups were defined based on the topology of the phylogenomic tree, namely, *Magnetospirillum* and *Phaeospirillum* (Group I); *Rhodospirillum, Pararhodospirillum, Novispirillum, Caenispirillum* and *Haematospirillum* (Group II); *Terasakiella, Thalassospira, Magnetospira* and *Magnetovibrio* (Group III); *Nisaea, Thalassobaculum, Oceanibaculum, Fodinicurvata* and *Rhodovibrio,* (Group IV); and *Azospirillum, Niveispirillum, Nitrospirillum, Skermanella, Rhodocista (Rhodospirillum centenum SW), Dongia, Elstera* and *Inquilinus* (Group V). The drawing pins in the nodes of Groups I, II and III indicate that these three groups have a common ancestor that contains both magnetosome synthesis (gray pushpin) and photosynthesis genes (pink pushpin)

Based on the phylogenomic tree, the members in the family Rhodospirillaceae can be separated into five main clades (Fig. 5): *Magnetospirillum* and *Phaeospirillum* (Group I); *Rhodospirillum, Pararhodospirillum, Novispirillum, Caenispirillum* and *Haematospirillum* (Group II); *Terasakiella, Thalassospira, Magnetospira* and *Magnetovibrio* (Group III); *Nisaea, Thalassobaculum, Oceanibaculum, Fodinicurvata* and *Rhodovibrio* (Group IV); and *Azospirillum, Niveispirillum, Nitrospirillum, Skermanella, Rhodocista* (*Rhodospirillum centenum* SW)*, Dongia, Elstera* and *Inquilinus* (Group V). Group I and Group II cluster together with Group III, while Group IV and Group V cluster closer (Fig. 5). Group I mainly consists of MTB, while most of the members from Group II are able to perform photosynthesis. In Group III, nonmagnetotactic *Thalassospira* clustered together with three MTB strains (genera *Terasakiella, Magnetospira* and *Magnetovibrio*). No MTB has been found in Group IV or Group V.

In the phylogenomic tree, the MTB *Magnetospirillum* strains and phototrophic bacteria *Rhodospirillum* strains are closely clustered, and in the photosynthesis branch, the *Phaeospirillum* genus clusters within the *Magnetospirillum* genus in Group I using concatenated genomic protein sequence alignment and clustering, which is in line with a previous 16S rRNA gene phylogenetic study [21]. Moreover, core genomic analysis was also conducted, and all predicted proteins from the 84 bacteria genomes described above were clustered into 409,598 total categories based on their protein sequences by OrthoMCL (Additional file 1: Figure S6, Additional file 4: Table S3). A high number of 2290 orthologous genes are shared by all five groups within the family Rhodospirillaceae. Interestingly, ancestor analysis based on the classified groups using Dollo parsimony method in COUNT program (Fig. 5, shown as drawing pins) indicates that both magnetosome synthesis and photosynthesis genes are present at the ancestral nodes of Groups I, II and III, while photosynthesis genes appeared at the ancestral nodes of all Rhodospirillaceae. This result provides a strong indication that both traits are likely vertically inherited rather than inherited through the HGT process.

**Fig. 6:**
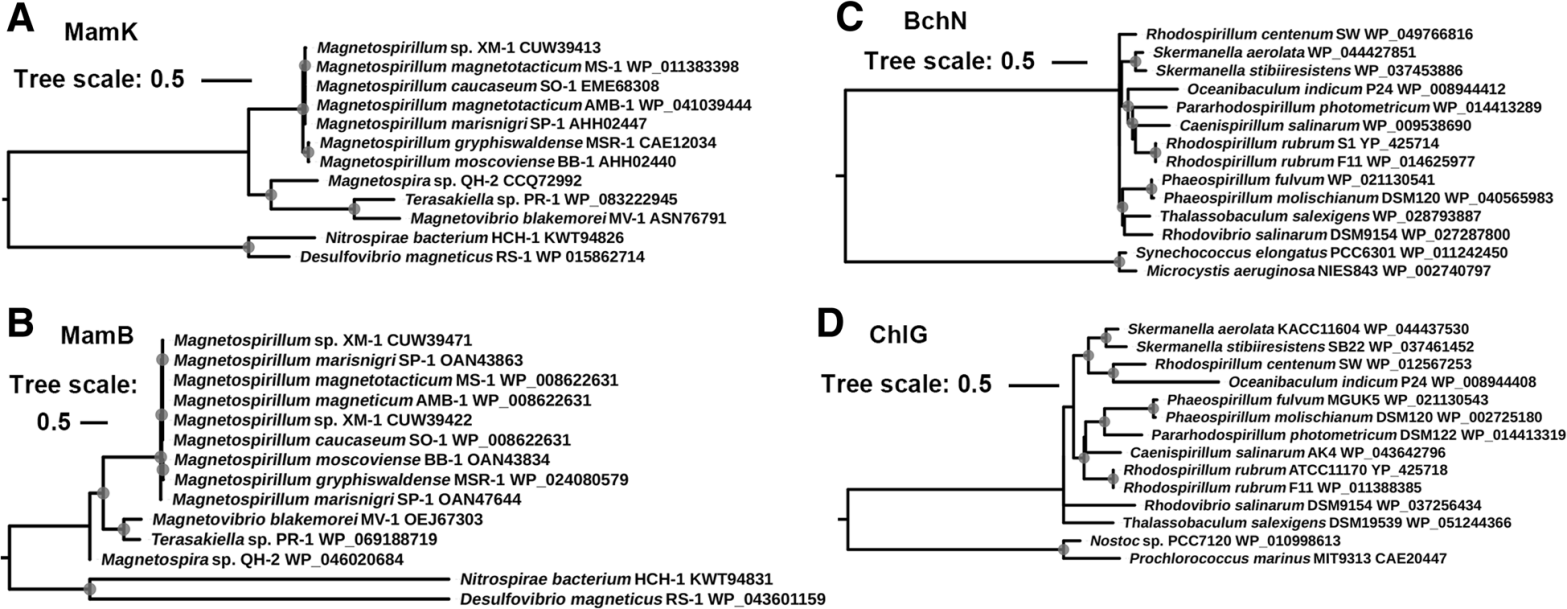
Phylogenetic trees based on MamK (**a**), MamB (**b**), BchN (**c**) and ChlG (**d**) protein sequences from the genomes of the family Rhodospirillaceae

To evaluate the independent phylogenetic relationships of MTB and phototrophic bacteria in Rhodospirillaceae, both conserved magnetosome-associated genes (*mam*K and *mam*B) and photosynthesis-related genes (*chl*G and *bch*N) were used as representatives for phylogenetic tree construction (Fig. 6). The proteins MamK and MamB are responsible for magnetosome chain formation and iron transportation during magnetosome synthesis, respectively, while the proteins ChlG and BchN are involved in bacteriochlorophyll/chlorophyll a synthesis and light-independent protochlorophyllide synthesis, respectively. These genes are specific to MTB or phototrophic bacteria, i.e., only MTB genomes harbored magnetosome synthesis genes, whereas only phototrophic bacteria genomes harbored photosynthesis genes. The topologies of the phylogenetic trees of both magnetosome synthesis and photosynthesis genes are reasonably consistent with the topology in the phylogenomic tree. These results suggest that MTB closely resemble phototrophic bacteria in this family and again indicate that magnetosome synthesis and photosynthesis abilities were likely vertically transferred during the evolution of the Rhodospirillaceae ancestor.

## Discussion

### The potential mechanism of MTB response to illumination

Bacterial phototaxis usually involves photosensing proteins and was first discovered in phototrophic organisms. Photosensing proteins function in the regulation of the expression of the photosynthesis machinery and protection of cells from harmful exposure to illumination [53]. Photoreceptor proteins were also found in nonphotosynthetic bacteria and functioned in signal transduction mostly for motility, biofilm forming, life cycle and the induction of carotene for protection against harmful light [54, 55]. The discoveries that MTB were also able to respond to blue and UV light were intriguing. Several photoreceptors were found in the uncultured MMP genomes [14]. It was hypothesized that, in environmental samples, a fraction of MMPs consistently display north-seeking behavior, which contrasts the expected swimming direction in the southern hemisphere, and this behavior was suggested to be regulated by light, stimulating the microorganism to move downwards to find suitable living environments [10].

Nevertheless, in most cases, MTB usually swim along the magnetic field against the oxygen concentration down to the surface sediments to occupy the OATZ [1, 2]. Moreover, *Magnetospirillum magneticum* AMB-1 was found to migrate towards visible light, while a similar phenomenon was observed in XM-1 swimming towards UVA radiation (Additional file 1: Figure S7) rather than moving away such as MMPs. The different phototaxis behaviors possessed by different MTB strains may be due to their distinct photoreceptors. The members of the *Magnetospirillum* clade, including the strains XM-1 and AMB-1, contain PHY domain photosensitive proteins that are considered to be red/far-red light-sensing bacteriophytochromes [54]. This photoreceptor may help *Magnetospirillum* to receive the photon signal for motility or potential physiological changes. XM-1 also contains a putative LOV/PAS-like domain-containing protein-coding gene that may help receive short wavelength light [54]. These *Magnetospirillum* strains usually live in aquatic environments and exhibit the ability to navigate towards sediment. The migration towards light may help them to move upwards to find better living environments that may contain trace oxygen. However, we cannot rule out that the light-dependent signal may respond to other physiological processes or only the redundant genes that their ancestor left behind. Moreover, other *Magnetospirillum* strains, such as MSR-1, BB-1 and QH-2, contain multiple photoreceptors, such as PYP, PHY and BLUF domain proteins, that respond to different wavelengths of the spectrum [53–55]. Thus, illumination may influence MTB behaviors or physiology in a more diverse way than previously thought. However, genetic experiments are needed to clearly illustrate the functions of these photoreceptors.

### Relationship between MTB and phototrophic bacteria within the Rhodospirillaceae family

In the family Rhodospirillaceae, the phototrophic bacteria *Rhodospirillum* and *Phaeospirillum* closely cluster with *Magnetospirillum* MTB in the phylogenomic tree (Fig. 5), and their central metabolic pathways and stress response systems are highly similar (Figs. 2, 4 and Additional file 1: Figure S4). Moreover, the successful transfer of the *Magnetospirillum gryphiswaldense* MGC into phototrophic *Rhodospirillum rubrum,* which enabled its magnetosome production ability [22], further indicates that they have the potential to hold both magnetotactic and phototrophic abilities. This genetically engineered organism was the first report of magnetosome-producing photosynthetic bacteria and consequently posed an interesting topic regarding whether the ancestor of Rhodospirillaceae bacteria was able to photosynthesis while mineralizing magnetic particles for navigation, but lost one or both abilities during evolutionary adaptive radiation in new niches, or whether divergent Rhodospirillaceae bacteria acquired one of these abilities via HGT during evolution. The photosynthesis ability within Rhodospirillaceae was generally believed to diverge from a phototrophic ancestor before the divergence of Proteobacteria [56, 57]. The ancestor analysis by COUNT program and phylogenetic tree of photosynthesis genes also confirmed that these genes might be vertically transferred in the family Rhodospirillaceae (Fig. 5). For the origin of microbial magnetotaxis and magnetosome biomineralization, arguments regarding the mono- or multiple-origin have been addressed by several studies, and more studies tend to support the hypothesis that MTB have only one common ancestor [24, 26, 58–60]. These hypotheses together might support the coexistence of magnetotaxis and photosynthesis within one cell in nature.

Gene loss, which is a mechanism for bacterial adaptation to different ecotypes in heterogeneous environments [61–63], might result in the divergence of this family. The evolutionary loss of traits can occur if traits are selected against or if a trait becomes redundant, and this is typically driven by the weakening or removal of selection pressures that are responsible for maintaining the trait [64, 65]. In this study, most of the MTB strains in the family Rhodospirillaceae contained MGC that was enriched with mobile elements and was easily deleted from the genome, especially in oxygen-rich environments [25]. Moreover, studies on aerobic phototrophic bacteria in eutrophic oceanic areas or rich organic media with prolonged growth could also allow the loss of phototrophic genes [66]. Therefore, if the original Rhodospirillaceae ancestor was equipped with both abilities, the ancestor may have diverged into phototrophic, magnetotactic and nonphototrophic nonmagnetotactic bacteria during niche differentiation.

### Geological implication

The possession of both magnetotaxis and phototrophic abilities might be evolutionarily beneficial for aquatic bacteria in early Earth’s history. The negative biological effects of ultraviolet radiation (UVR) in the Archean era have been estimated to be approximately three-fold higher than the present time [67]. Hence, without the ozone layer as a natural protection filtering UVR [68], the photosynthesis bacteria may suffer strong UV-induced damage living in the light penetration zone in the upper layer of the aquatic environment. To avoid deleterious or lethal UVR while still acquiring enough illumination for energy, it is reasonable to assume that these ancient phototrophic microbes may have developed the simple mechanism of navigation, i.e., magnetotaxis. This allowed them to swim downwards away from the strong UVR gradient and helped them find the ultraviolet tolerance photosynthesis zone (UTPZ) instead of the OATZ in the present time (Additional file 1: Figure S8).

## Conclusion

With increasing evidence that MTB are able to positively or negatively respond to light, the knowledge of why MTB can react to light, how they mediate damage induced by light, and the evolutionary relationship between MTB and phototrophic bacteria are of great interest to microbiologists. In the current study, by utilizing the sequenced genomes of *Magnetospirillum*, phototrophic *Rhodospirillum* and other bacteria within the Rhodospirillaceae family, analyses of their photoreceptors, oxidative stress systems, DNA damage repair abilities and phylogenomics were conducted to answer these questions. Both MTB and phototrophic bacteria contain photosensitive domain proteins. The oxidative stress and DNA damage repair systems of the family Rhodospirillaceae are complex but do not show significant differences. *Magnetospirillum* species closely cluster with the phototrophic bacteria *Rhodospirillum* and *Phaeospirillum* in the phylogenomic tree. The topology of the phylogenomic tree is similar to both phylogenetic tree topologies of the magnetosome synthesis and photosynthesis genes. These results indicate that MTB, phototrophic and nonphototrophic nonmagnetic bacteria from the Rhodospirillaceae family likely evolved vertically with magnetosome synthesis and photosynthesis genes. The ancestor of Rhodospirillaceae was likely a magnetotactic phototrophic bacteria, however, gain or loss of magnetotaxis and phototrophic abilities might have occurred during the evolution of ancestral Rhodospirillaceae lineages.

## Methods

### The XM-1 genome analysis and the selection of the Rhodospirillaceae genomes

The MTB strain XM-1 was isolated from Xi’an city moat in Northwest China, cultured in optimized XM-C medium [28] and then sequenced by Illumina HiSeq 2000, and the gaps were closed by PCR [29]. The XM-1 genome is available in the NCBI repository under Project Number PRJEB11958, https://www.ncbi.nlm.nih.gov/bioproject/PRJEB11958/. The genome was annotated at MicroScope MaGe [69], and the genomic colinearity of XM-1 and other MTB were analyzed by the program mummer 3.0 [70]. Average nucleotide identity (ANI) and average amino acid identity (AAI) were also calculated with the ANI/AAI calculator [71].

All genomes of cultivated bacteria within the family Rhodospirillaceae were downloaded from NCBI (https://www.ncbi.nlm.nih.gov), and genomes with low quality (contigs > 500) were removed. A total of 84 high-quality genomes (Additional file 2: Table S1) were obtained, and the metabolic capacities were analyzed with the KEGG database [72].

### Phylogenetic and phylogenomic trees calculation

The representative 16S rRNA genes of MTB and the Rhodospirillaceae strains were downloaded from NCBI. The phylogenetic tree was constructed using MEGA6.06 [73]. For the phylogenomic tree, MTB genomes outside Rhodospirillaceae, as well as Aquificae and Cyanobacteria genomes, were also downloaded from NCBI as out groups. All genomes within the Rhodospirillaceae family and out groups used in the current study were first aligned by PhyloPhlAn software [74], and the phylogenomic tree was built by the RAxML method [75] with the --auto-prot = bic option using the PROTGAMMAAUTO model with a bootstrap value of 1000.

### Comparative genomic analysis of Rhodospirillaceae genomes

The orthologous protein families from the 84 selected genomes were identified using OrthoMCL [76] with a BLASTp E-value threshold of 10^− 5^, a 50% coverage cutoff with 30% identity and a default MCL inflation parameter of 1.5. For oxidative stress and iron homeostasis analysis, PeroxiBase (http://peroxibase.toulouse.inra.fr/) and one previously reported iron transporter database [77] were modified and used to search for the related genes using BLASTp with an E-value of 10^− 20^ and 75% coverage with 30% identity as a cutoff. The resulting protein sequences were BLASTp searched against the nonredundant NCBI protein database for confirmation. Only those sequences that reported top hits to the correct functions were considered in our analyses. The ambiguous sequences that contained specific functional domains were checked manually in the NCBI Conserved Domains database. To address the evolution histories of magnetosome synthesis and phototrophic abilities from the family Rhodospirillaceae, ancestral family sizes were inferred using the program COUNT with Dollo parsimony algorithm [78]. This approach strictly prohibits multiple gains of genes and allows reconstructing gene gain and loss events at both observed species and potential ancestors (leaves and nodes on the phylogenetic tree).

## Additional files

Additional file 1: **Figure S1.** Circular diagrams of the *Magnetospirillum* sp. XM-1 chromosome and plasmid show the relevant genome features. **Figure S2.** Colinearity plot shows the comparison of genome from the *Magnetospirillum* sp. XM-1 and *Magnetospirillum magneticum* AMB-1. **Figure S3.** (A) The comparison of iron related genes. (B) Summary of iron homeostasis features identified in the genomes. (C) Sketch of iron homeostasis systems in the Rhodospirillaceae family. **Figure S4.** The comparison of DNA damages repair genes. **Figure S5.** The comparison of the percentage of DNA damage repair gene from MTB and non-MTB. **Figure S6.** (A) Vann diagram of core, dispensable and specific genes from five groups. (B) Vann diagram of core, dispensable and specific genes. Figure S7. MTB strain XM-1 swam towards the UVA radiation and accumulated at the illuminate side of the quartz bottle. **Figure S8.** Sketch map shows the strategy of possible photosynthesis magnetotactic bacteria in Archean Eon when surface UV radiation was high. Magnetotaxis could help them to swim down to the ultraviolet tolerance photosynthesis zone (UTPZ) to avoid lethal doses of irradiation while harvest enough light. (PDF 3745 kb)

Additional file 2: **Table S1.** General features of the XM-1 genome compared with other representative MTB genomic sequences from Rhodospirillaceae (PDF 64 kb)

Additional file 3: **Table S2.** The bacteria genomes used in this study (PDF 105 kb)

Additional file 4: **Table S3 A.** The core inventory shared by the family Rhodospirillaceae; **Table S3 B.** The core genes among MTB, phototrophic bacteria, non-phototrophic non-magnetotactic bacteria within Rhodospirillaceae and other MTB that are not related to the family Rhodospirillaceae (XLSX 178 kb)

Additional file 5: **Table S4 A.** Oxidative related genes from the Rhodospirillaceae members; **Table S4 B.** Iron related genes from the Rhodospirillaceae members; **Table S4 C** DNA repair related genes from the Rhodospirillaceae members (XLSX 459 kb)

## Abbreviations

AAI: Average amino acid identity
ANI: Average nucleotide identity
BER: Base pair excision repair
BLUF: Blue-light using FAD
CBB cycle: Calvin–Benson–Bassham cycle
DSR: Dissimilatory sulfite reductase
GGDEF: Gly-Gly-Asp-Glu-Phe amino-acid motif
HGT: Horizontal gene transfer
LOV: Light-oxygen-voltage photoreceptor
MGC: Magnetosome gene cluster
MMP: Multicellular magnetotactic prokaryote
MTB: Magnetotactic bacteria
NER: Nucleotide excision repair
OATZ: Oxic-anoxic transition zone
PHY: Red/far red light sensitive phytochrome
PYP: Photoactive yellow protein
ROS: Reactive oxygen species
rTCA cycle: reductive tricarboxylic acid cycle
RubisCO: Ribulose-1,5-bisphosphate carboxylase-oxygenase
UTPZ: Ultraviolet tolerance photosynthesis zone
UVR: Ultraviolet radiation
