# Supplementary material for: Genomic evidence of the illumination response mechanism and evolutionary history of magnetotactic bacteria within the Rhodospirillaceae family
Source: BMC Genomics. 2019 May 22;20:407. doi: 10.1186/s12864-019-5751-9 (PMC6532209; doi:10.1186/s12864-019-5751-9)
Supplement: Supplementary file 1 — Figure S1. Circular diagrams of the Magnetospirillum sp. XM-1 chromosome and plasmid show the relevant genome features. Figure S2. Colinearity plot shows the comparison of genome from the Magnetospirillum sp. XM-1 and Magnetospirillum magneticum AMB-1. Figure S3. (A) The comparison of iron related genes. (B) Summary of iron homeostasis features identified in the genomes. (C) Sketch of iron homeostasis systems in the Rhodospirillaceae family. Figure S4. The comparison of DNA damages repair genes. Figure S5. The comparison of the percentage of DNA damage repair gene from MTB and non-MTB. Figure S6. (A) Vann diagram of core, dispensable and specific genes from five groups. (B) Vann diagram of core, dispensable and specific genes. Figure S7. MTB strain XM-1 swam towards the UVA radiation and accumulated at the illuminate side of the quartz bottle. Figure S8. Sketch map shows the strategy of possible photosynthesis magnetotactic bacteria in Archean Eon when surface UV radiation was high. Magnetotaxis could help them to swim down to the ultraviolet tolerance photosynthesis zone (UTPZ) to avoid lethal doses of irradiation while harvest enough light. (PDF 3745 kb) [file 12864_2019_5751_MOESM1_ESM.pdf]

Genomic evidence of the illumination response mechanism and evolutionary history of magnetotactic bacteria within the Rhodospirillaceae family

Yinzhao Wang[1,2,5*], Giorgio Casaburi[3], Wei Lin[2], Ying Li[4], Fengping Wang[1] and Yongxin Pan[2,5]

[1]State Key Laboratory of Microbial Metabolism, School of Life Sciences and Biotechnology, Shanghai Jiao Tong University, Shanghai, China; [2]Institute of Geology and Geophysics, Chinese Academy of Sciences, Beijing, China 100029; [3]Departments of Microbiology and Cell Science, Space Life Sciences Laboratory, University of Florida, Merritt Island, FL 32953, USA; [4]State Key Laboratory of Agrobiotechnology and College of Biological Sciences, China Agricultural University, Beijing 100193, China; [5]University of Chinese Academy of Sciences, Beijing, China 100049;

Correspondence author:
Yinzhao Wang
Shanghai Jiao Tong University
E-mail: wyz@sjtu.edu.cn

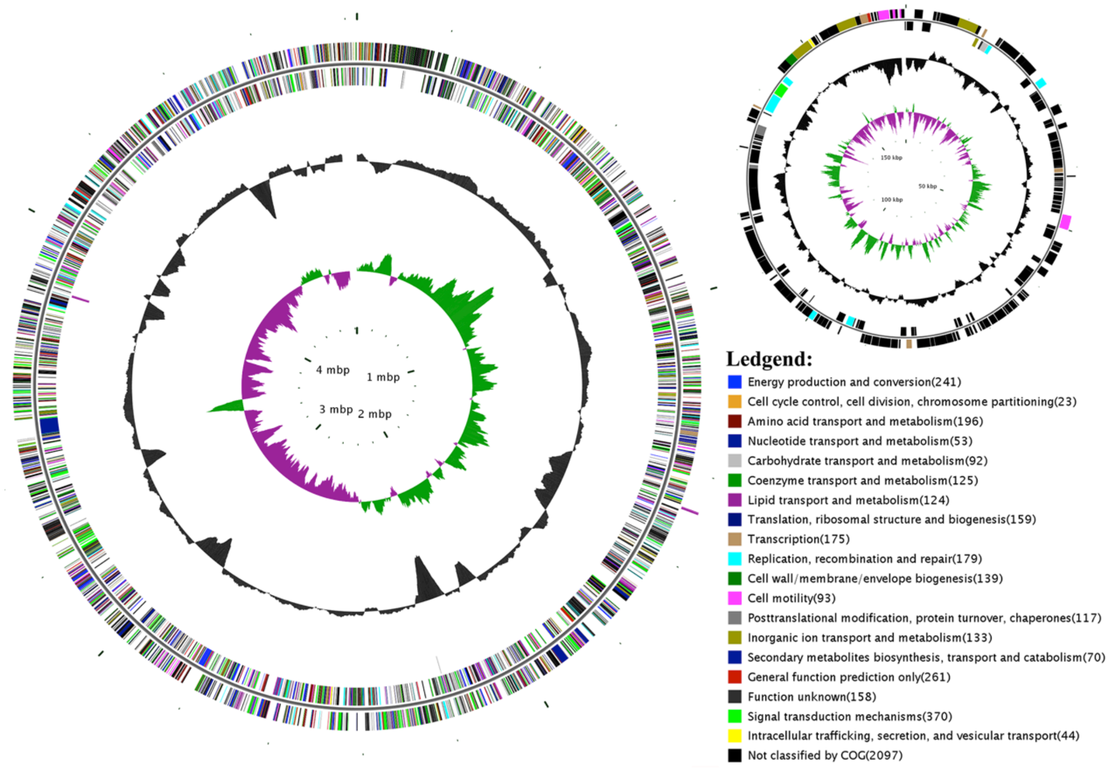

Figure S1. Circular diagrams of the *Magnetospirillum* sp. XM-1 chromosome and plasmid show the relevant genome features. Circles in chromosome contains information of position (in megabases); forward and reverse strand CDSs (colours indicating the assigned COG classes); G + C content (red indicates higher G + C compared with the chromosome average G + C content and green indicates lower G + C content); GC skew.

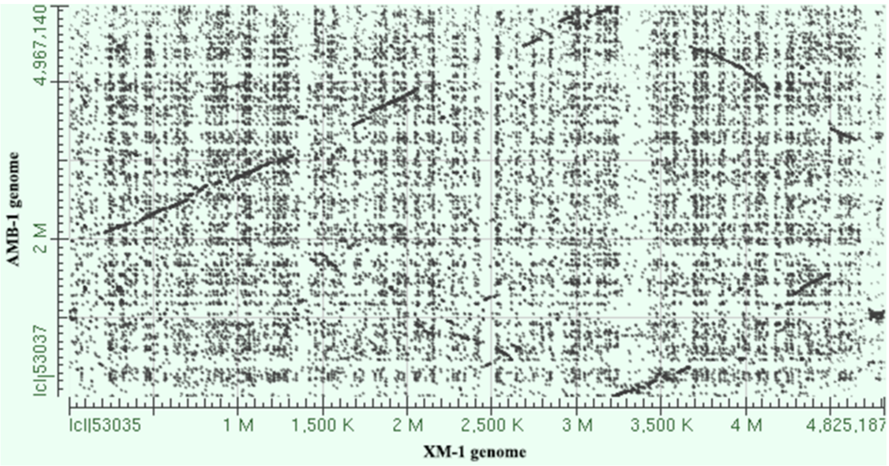

Figure S2. Colinearity plot shows the comparison of genome from the *Magnetospirillum* sp. XM-1 and *Magnetospirillum magneticum* AMB-1.

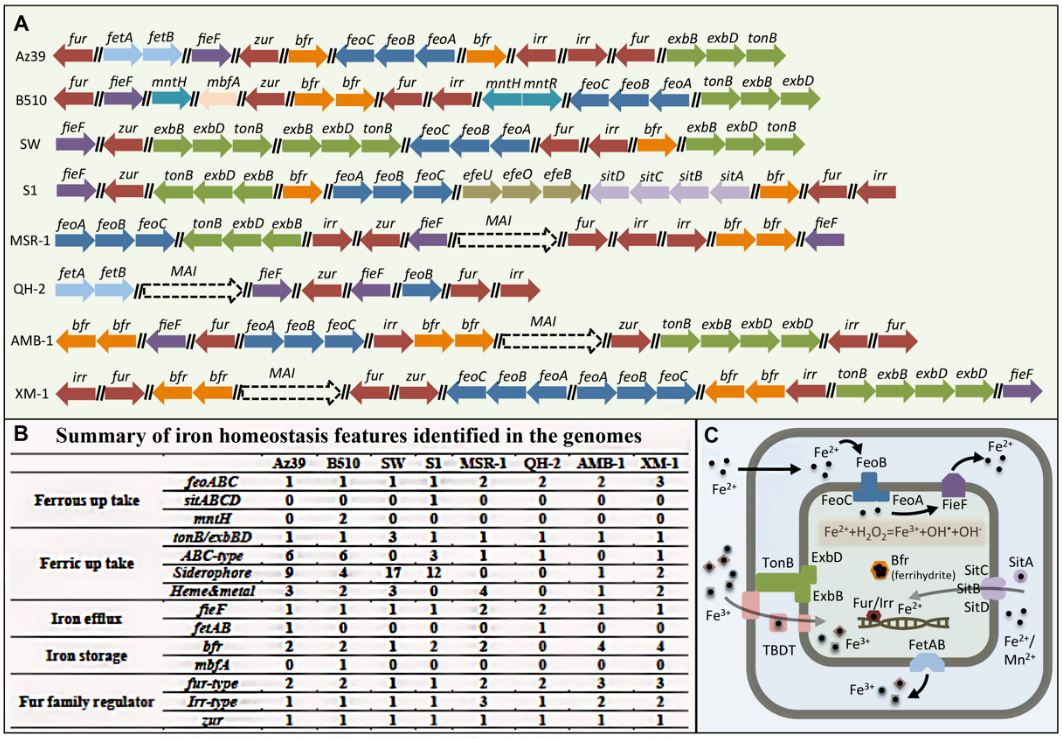

		Az39	B510	SW	S1	MSR-1	QH-2	AMB-1	XM-1
Ferrous up take	*feoABC*	1	1	1	1	2	2	2	3
	sitABCD	0	0	0	1	0	0	0	0
	mntH	0	2	0	0	0	0	0	0
Ferric up take	*tonB/exbBD*	1	1	3	1	1	1	1	1
	ABC-type	6	6	0	3	1	1	0	1
	Siderophore	9	4	17	12	0	0	1	2
	Heme&metal	3	2	3	0	4	0	1	2
Iron efflux	*fieF*	1	1	1	1	2	2	1	1
	fetAB	1	0	0	0	0	1	0	0
Iron storage	*bfr*	2	2	1	2	2	0	4	4
	mbfA	0	1	0	0	0	0	0	0
Fur family regulator	*fur-type*	2	2	1	1	2	2	3	3
	Irr-type	1	1	1	1	3	1	2	2
	zur	1	1	1	1	1	1	1	1

Figure S3. (A) The comparison of iron related genes of the MTB strains XM-1, AMB-1, MSR-1 and QH-2, phototrophic strains S1 and SW, non-magnetic and non-phototrophic strains Az39 and B510 from the Rhodospirillaceae family. (B) Summary of iron homeostasis features identified in the genomes. (C) Sketch of iron homeostasis systems in the Rhodospirillaceae family.

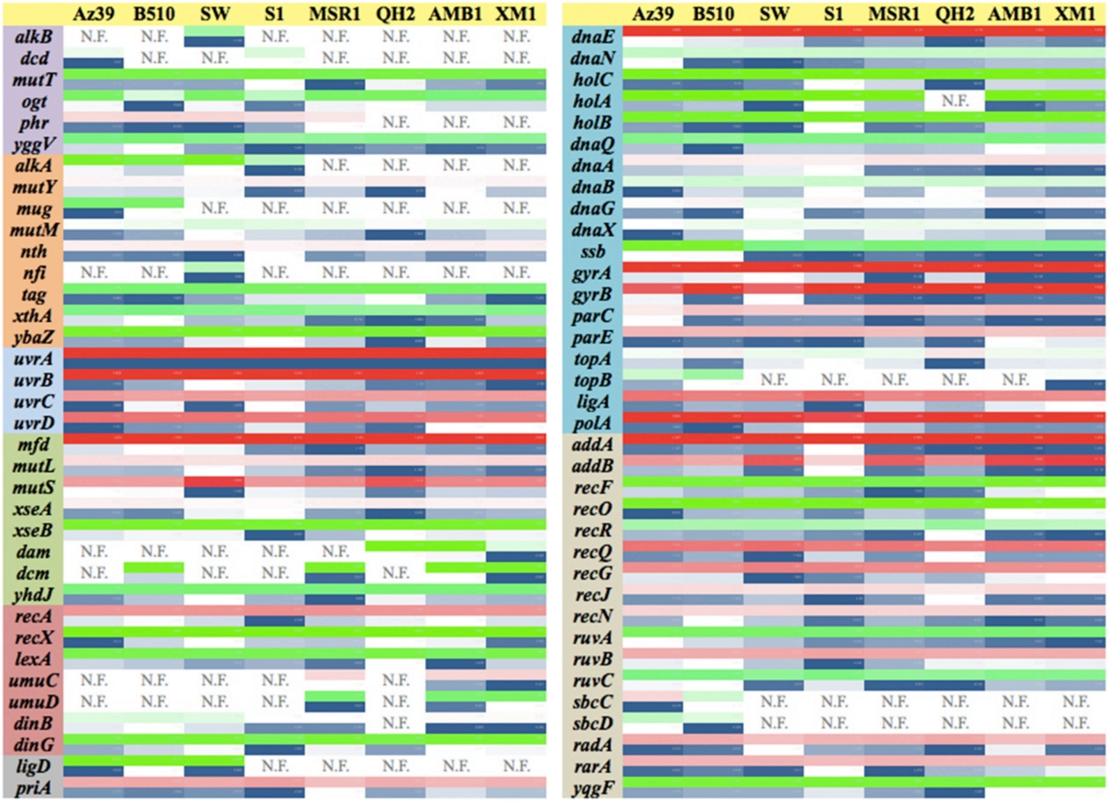

Figure S4. The comparison of DNA damages repair genes of the MTB strains XM-1, AMB-1, MSR-1 and QH-2, phototrophic strains S1 and SW, non-magnetic and non-phototrophic strains Az39 and B510 from the Rhodospirillaceae family. The gene names are listed at the left sides of the diagram and the background colors of the genes referred to direct reversal (purple), base excision repair (orange), nucleotide excision repair (blue), mismatch repair (green), SOS repair (red), double-string break repair (gray), DNA replication (cyan) and DNA recombination (brown). The diagram shows the presence (color bar) and absence (N.F.) of the DNA damage repair gene within different bacteria. The upper color bars in the heat map represent the identities between the DNA damages repair gene and the green means the identity is low while red refers to high. The grayscales of lower color bar represent the identity comparison within each group.

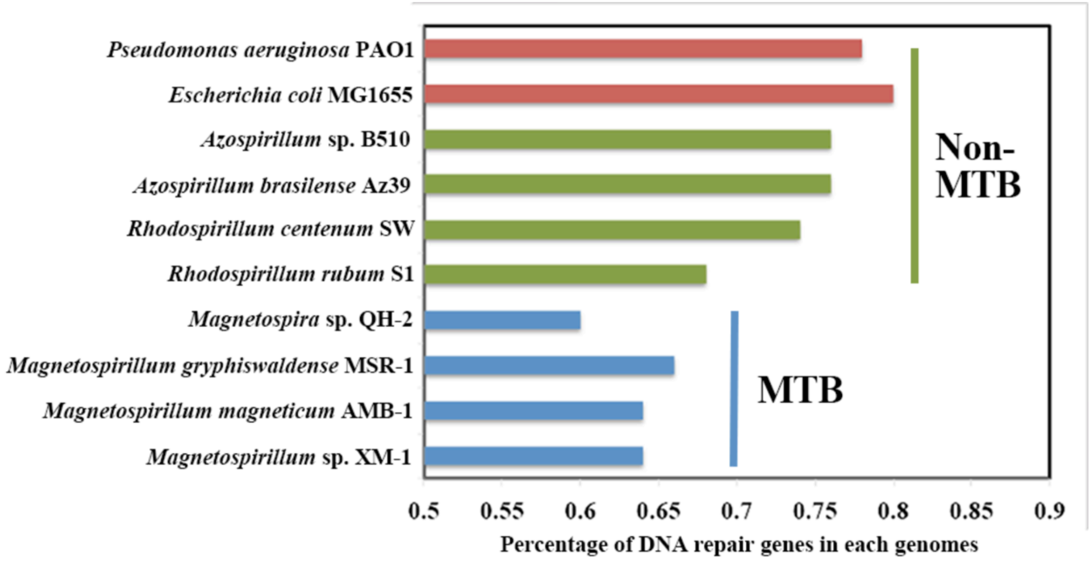

Figure S5. The comparison of the percentage of DNA damage repair gene from MTB and non-MTB.

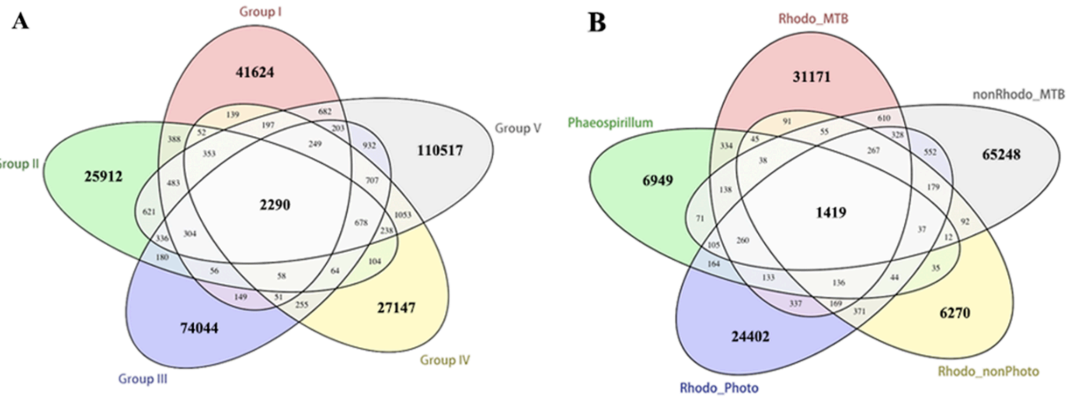

Figure S6. (A) Vann diagram of core, dispensable and specific genes from five groups. (B) Vann diagram of core, dispensable and specific genes from MTB within the family Rhodospirillaceae (Rhodo_MTB), Phaeospirillum, phototrophic bacteria within Rhodospirillaceae (Rhodo_Photo), non-phototrophic bacteria within Rhodospirillaceae (Rhodo_nonPhoto) and other MTB that are not related to the family Rhodospirillaceae (nonRhodo_MTB).

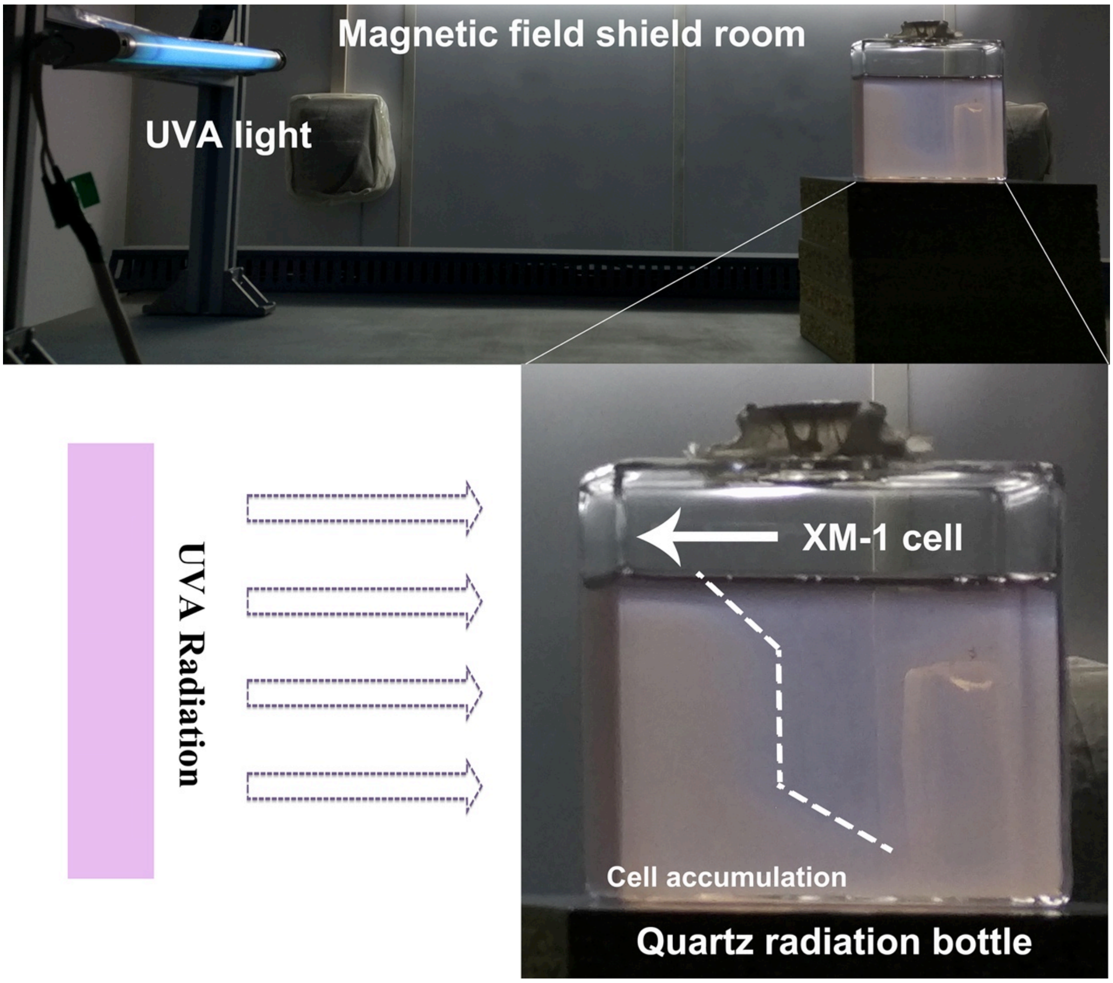

Figure S7. MTB strain XM-1 swam towards the UVA radiation and accumulated at the illuminate side of the bottle.

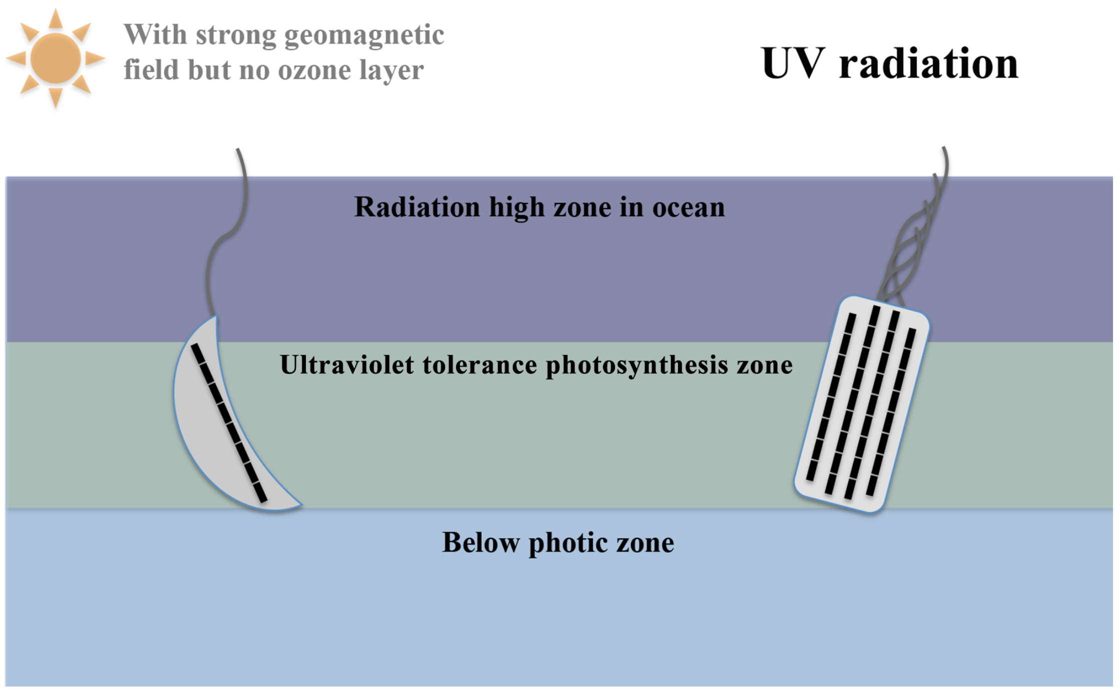

Figure S8. Sketch map shows the strategy of possible photosynthesis magnetotactic bacteria in Archean eon when surface UV radiation was high. Magnetotaxis could help them to swim down to the ultraviolet tolerance photosynthesis zone to avoid lethal doses of irradiation while harvest enough light.
