# Supplementary material for: Genomic evidence of the illumination response mechanism and evolutionary history of magnetotactic bacteria within the Rhodospirillaceae family
Source: BMC Genomics. 2019 May 22;20:407. doi: 10.1186/s12864-019-5751-9 (PMC6532209; doi:10.1186/s12864-019-5751-9)
Supplement: Supplementary file 2 — Table S1. General features of the XM-1 genome compared with other representative MTB genomic sequences from Rhodospirillaceae (PDF 64 kb) [file 12864_2019_5751_MOESM2_ESM.pdf]

Table S1 General features of the XM-1 genome compared with other representative MTB genomic sequences from Rhodospirillaceae

	***Magnetospirillum* sp. XM-1**		***Magnetospirillum Magneticum* AMB-1**	***Magnetospirillum gryphiswaldense* MSR-1**	***Magnetospira* sp. QH-2**	
	Chromosome	Plasmid	Chromosome	Chromosome	Chromosome	Plasmid
Size	4 825 187	167 290	4 967 148	4 365 796	4 020 900	31 063
GC	65.64%	66.48%	65.1%	63.28%	59.5%	54.8%
CDS	4 550	186	4 561	4 261	3 794	37
rRNA	2	-	2	2	3	-
tRNA	53	-	46	50	47	-
Prophage	4	-	12	7	0	-
GI	28	-	48	23	12	-
