# Supplementary material for: Genomic evidence of the illumination response mechanism and evolutionary history of magnetotactic bacteria within the Rhodospirillaceae family
Source: BMC Genomics. 2019 May 22;20:407. doi: 10.1186/s12864-019-5751-9 (PMC6532209; doi:10.1186/s12864-019-5751-9)
Supplement: Supplementary file 3 — Table S2. The bacteria genomes used in this study (PDF 105 kb) [file 12864_2019_5751_MOESM3_ESM.pdf]

Table S2 The bacteria genomes used in this study

Strain	Scaffold	Contig	Complete
**Rhodospirillaceae**			
Azospirillum brasilense FP2	413	413	No
Azospirillum brasilense Sp245	7	67	No
Azospirillum brasilense SP7	6	6	Yes
Azospirillum brasilense Az39	6	6	Yes
Azospirillum halopraeferens DSM 3675	50	56	No
Azospirillum humicireducens SgZ-5	1	1	Yes
Azospirillum lipoferum 4B	7	7	Yes
Azospirillum sp. B506	1143	1144	No
Azospirillum sp. B510	7	7	Yes
Azospirillum sp. CAG:239	109	160	No
Azospirillum sp. CAG:260	83	118	No
Azospirillum thiophilum BV-S	8	8	Yes
Azospirillum thiophilum DSM 21654	11	11	No
Caenispirillum salinarum AK4	61	61	No
Candidatus Endolissoclinum faulkneri L2	1	1	Yes
Candidatus Endolissoclinum faulkneri L5	1	1	Yes
Dongia sp. URHE0060	19	22	No
Elstera litoralis Dia-1	1012	1012	No
Fodinicurvata fenggangensis DSM 21160	37	37	No
Fodinicurvata sediminis DSM 21159	20	20	No
Haematospirillum jordaniae H5569	3	3	Yes
Inquilinus limosus DSM 16000	64	74	No
Inquilinus limosus MP06	1186	1186	No
Magnetovibrio blakemorei MV-1	91	91	No
Magnetospira sp. QH-2	2	2	Yes
Magnetospirillum caucaseum SO-1	236	236	No
Magnetospirillum gryphiswaldense MSR-1	1	1	Yes
Magnetospirillum magneticum AMB-1	1	1	Yes
Magnetospirillum magnetotacticum MS-1	36	36	No
Magnetospirillum marisnigri SP-1	131	132	No
Magnetospirillum moscoviense BB-1	207	207	No
Magnetospirillum xidanesis XM-1	2	2	Yes
Magnetospirillum sp. 64120	58	58	No
Nisaea denitrificans DSM 18348	20	22	No
Nitrospirillum amazonense Y2	1014	1014	No
Niveispirillum irakense DSM 11586	20	30	No
Novispirillum itersonii ATCC 12639	34	39	No
Oceanibaculum indicum P24	71	71	No
Oceanibaculum pacificum MCCC 1A02656	181	181	No

Pararhodospirillum photometricum DSM 122	1	1	Yes
Phaeospirillum fulvum MGU-K5	178	178	No
Phaeospirillum molischianum DSM 120	61	61	No
Rhodocista centenaria SW	1	1	Yes
Rhodospirillaceae bacterium BRH_c57	274	274	No
Rhodospirillaceae bacterium CCH5-H10	892	892	No
Rhodospirillum rubrum F11	1	1	Yes
Rhodospirillum rubrum S1 ATCC 11170	2	2	Yes
Rhodovibrio salinarum DSM 9154	1	3	Yes
Skermanella aerolata KACC 11604	276	276	No
Skermanella stibiiresistens SB22	190	190	No
Terasakiella sp. PR1	48	48	No
Thalassobaculum salexigens DSM 19539	12	17	No
Thalassospira australica NP3b2	32	32	No
Thalassospira lucentensis MCCC 1A00383	22	22	No
Thalassospira lucentensis MCCC 1A02072	25	25	No
Thalassospira permensis NBRC 106175	72	72	No
Thalassospira profundimaris WP0211	28	28	No
Thalassospira sp. HJ	22	22	No
Thalassospira sp. MCCC 1A01148	24	24	No
Thalassospira sp. MCCC 1A02491	33	33	No
Thalassospira sp. MCCC 1A02898	14	14	No
Thalassospira sp. Nap_22	301	301	No
Thalassospira sp. PB8B	34	34	No
Thalassospira tepidiphila MCCC 1A03514	27	27	No
Thalassospira xiamenensis M-5	2	2	Yes
Thalassospira xiamenensis MCCC 1A02795	30	30	No
Thalassospira xiamenensis MCCC 1A03005	62	62	No
Thalassospira xiamenensis MCCC 1A03042	72	72	No
Tistrella mobilis KA081020-065	1	4	Yes
Tistrella mobilis MCCC 1A02139	233	233	No
**Other magnetotactic bacteria**			
Candidatus Magnetobacterium bavaricum TM-1	2751	2752	No
Candidatus Magnetobacterium casensis MYR-1	70	70	No
Candidatus Magnetoglobus multicellularis Araruama	3705	3705	No
Candidatus Magnetomorum sp. HK-1	3036	3036	No
Candidatus Magnetoovum chiemensis CS-04	1019	1019	No
Candidatus Omnitrophus magneticus SKK-01	656	656	No
Desulfovibrio magneticus RS-1	3	3	Yes
Desulfovibrio magneticus MBC34	489	489	No
Desulfovibrio magneticus IFRC170	10	10	No
Magnetococcus marinus MC-1	1	1	Yes
Magnetofaba australis IT-1	21	21	No

Magnetococcus massalia MO-1	2	2	Yes
Nitrospirae bacterium HCH-1	152	152	No
**Representative cyanobacteria**			
Microcystis aeruginosa NIES-843	1	1	Yes
Synechococcus elongatus PCC 6301	2	2	Yes
Microcystis panniformis FACHB-1757	1	1	Yes
Cyanobacterium stanieri PCC 7202	1	1	Yes
Synechococcus sp. CC9902	7	7	Yes
Synechococcus sp. CC9605	1	1	Yes
Geminocystis sp. NIES-3708	1	1	Yes
Rivularia sp. PCC 7116	3	3	Yes
Halothece sp. PCC 7418	1	1	Yes
Prochlorococcus marinus MIT9313	1	1	Yes
Prochlorococcus marinus CCMP1375	1	1	Yes
Prochlorococcus sp. MIT0801	1	1	Yes
Leptolyngbya boryana PCC6306	5	19	No
Nostoc sp. PCC7120	1	1	Yes
Microcoleus sp. PCC 7113	10	10	Yes
